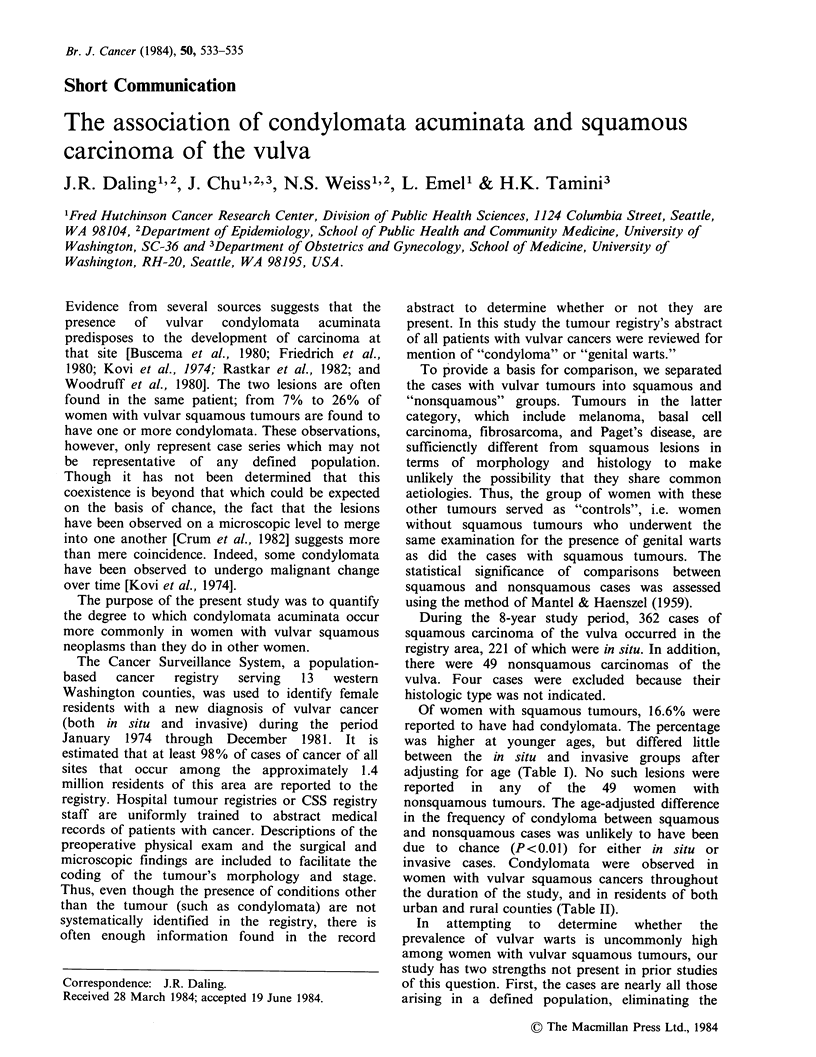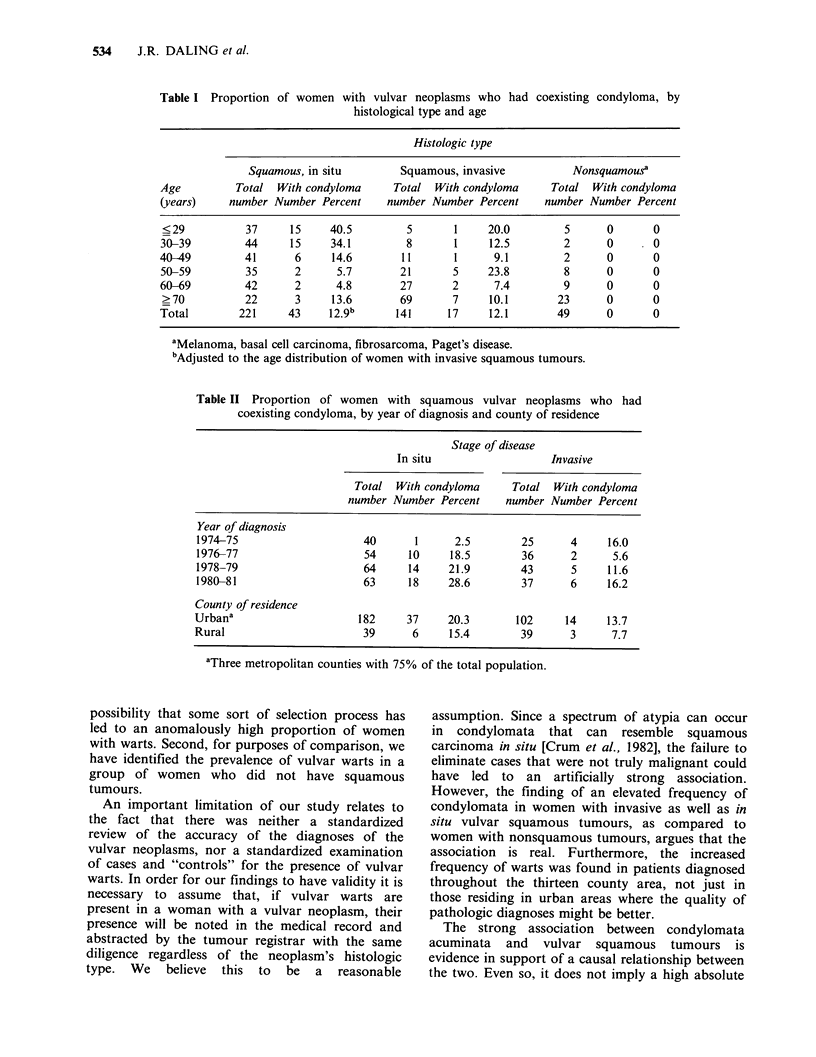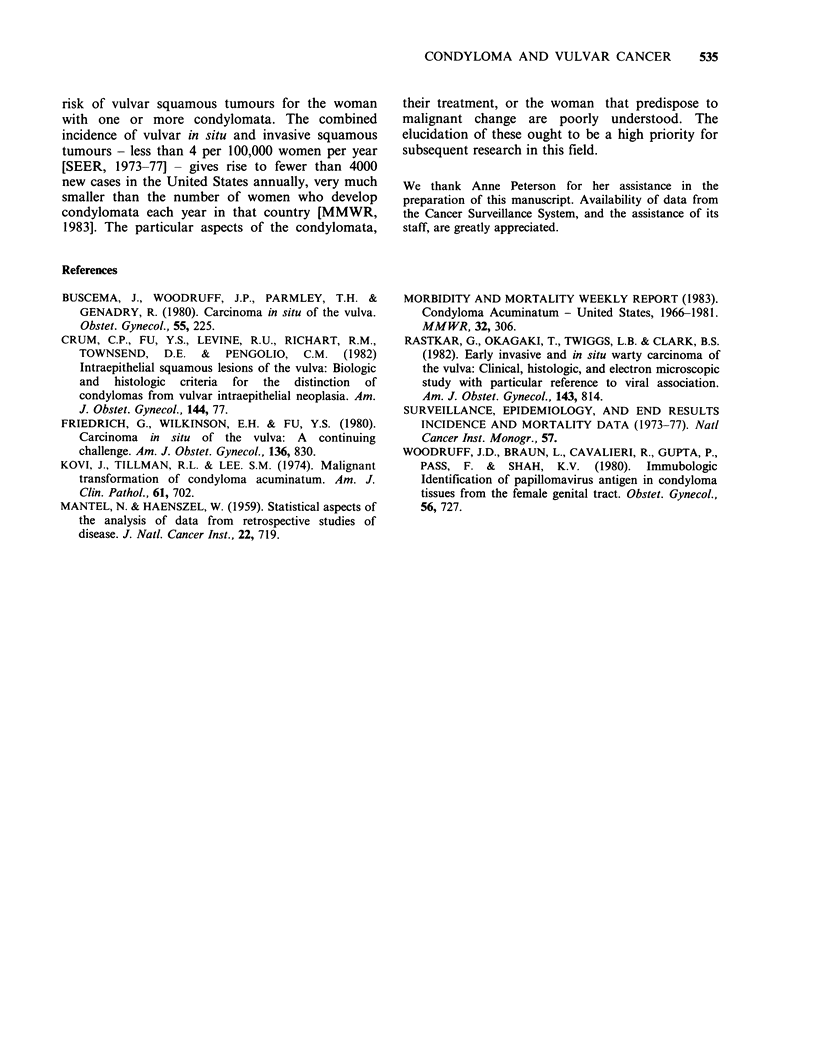# The association of condylomata acuminata and squamous carcinoma of the vulva.

**DOI:** 10.1038/bjc.1984.211

**Published:** 1984-10

**Authors:** J. R. Daling, J. Chu, N. S. Weiss, L. Emel, H. K. Tamini


*Br. J. Cancer* (1984), **50**, 533–535

### Short Communication

### The association of condylomata acuminata and squamous carcinoma of the vulva

J.R. Daling[1,2], J. Chu[1,2,3], N.S. Weiss[1,2], L. Emel[1] & H.K. Tamini[3]

[1]*Fred Hutchinson Cancer Research Center, Division of Public Health Sciences, 1124 Columbia Street, Seattle, WA 98104,* [2]*Department of Epidemiology, School of Public Health and Community Medicine, University of Washington, SC-36 and* [3]*Department of Obstetrics and Gynecology, School of Medicine, University of Washington, RH-20, Seattle, WA 98195, USA.*

Evidence from several sources suggests that the presence of vulvar condylomata acuminata predisposes to the development of carcinoma at that site [Buscema *et al.*, 1980; Friedrich *et al.*, 1980; Kovi *et al., 1974;* Rastkar *et al.*, 1982; and Woodruff *et al.*, 1980]. The two lesions are often found in the same patient; from 7% to 26% of women with vulvar squamous tumours are found to have one or more condylomata. These observations, however, only represent case series which may not be representative of any defined population. Though it has not been determined that this coexistence is beyond that which could be expected on the basis of chance, the fact that the lesions have been observed on a microscopic level to merge into one another [Crum *et al.*, 1982] suggests more than mere coincidence. Indeed, some condylomata have been observed to undergo malignant change over time [Kovi *et al.*, 1974].

The purpose of the present study was to quantify the degree to which condylomata acuminata occur more commonly in women with vulvar squamous neoplasms than they do in other women.

The Cancer Surveillance System, a population-based cancer registry serving 13 western Washington counties, was used to identify female residents with a new diagnosis of vulvar cancer (both *in situ* and invasive) during the period January 1974 through December 1981. It is estimated that at least 98% of cases of cancer of all sites that occur among the approximately 1.4 million residents of this area are reported to the registry. Hospital tumour registries or CSS registry staff are uniformly trained to abstract medical records of patients with cancer. Descriptions of the preoperative physical exam and the surgical and microscopic findings are included to facilitate the coding of the tumour's morphology and stage. Thus, even though the presence of conditions other than the tumour (such as condylomata) are not systematically identified in the registry, there is often enough information found in the record abstract to determine whether or not they are present. In this study the tumour registry's abstract of all patients with vulvar cancers were reviewed for mention of "condyloma" or "genital warts."

To provide a basis for comparison, we separated the cases with vulvar tumours into squamous and "nonsquamous" groups. Tumours in the latter category, which include melanoma, basal cell carcinoma, fibrosarcoma, and Paget's disease, are sufficienctly different from squamous lesions in terms of morphology and histology to make unlikely the possibility that they share common aetiologies. Thus, the group of women with these other tumours served as "controls", i.e. women without squamous tumours who underwent the same examination for the presence of genital warts as did the cases with squamous tumours. The statistical significance of comparisons between squamous and nonsquamous cases was assessed using the method of Mantel & Haenszel (1959).

During the 8-year study period, 362 cases of squamous carcinoma of the vulva occurred in the registry area, 221 of which were *in situ*. In addition, there were 49 nonsquamous carcinomas of the vulva. Four cases were excluded because their histologic type was not indicated.

Of women with squamous tumours, 16.6% were reported to have had condylomata. The percentage was higher at younger ages, but differed little between the *in situ* and invasive groups after adjusting for age (Table I). No such lesions were reported in any of the 49 women with nonsquamous tumours. The age-adjusted difference in the frequency of condyloma between squamous and nonsquamous cases was unlikely to have been due to chance ($P<0.01$) for either *in situ* or invasive cases. Condylomata were observed in women with vulvar squamous cancers throughout the duration of the study, and in residents of both urban and rural counties (Table II).

In attempting to determine whether the prevalence of vulvar warts is uncommonly high among women with vulvar squamous tumours, our study has two strengths not present in prior studies of this question. First, the cases are nearly all those arising in a defined population, eliminating the

Correspondence: J.R. Daling.
Received 28 March 1984; accepted 19 June 1984.

© The Macmillan Press Ltd., 1984

**Table I** Proportion of women with vulvar neoplasms who had coexisting condyloma, by histological type and age

| | Histologic type | | | | | | | | |
|---|---|---|---|---|---|---|---|---|---|
| | *Squamous*, in situ | | | Squamous, invasive | | | *Nonsquamous*[a] | | |
| *Age (years)* | *Total number* | *With condyloma Number* | *With condyloma Percent* | *Total number* | *With condyloma Number* | *With condyloma Percent* | *Total number* | *With condyloma Number* | *With condyloma Percent* |
| ≦29 | 37 | 15 | 40.5 | 5 | 1 | 20.0 | 5 | 0 | 0 |
| 30–39 | 44 | 15 | 34.1 | 8 | 1 | 12.5 | 2 | 0 | 0 |
| 40–49 | 41 | 6 | 14.6 | 11 | 1 | 9.1 | 2 | 0 | 0 |
| 50–59 | 35 | 2 | 5.7 | 21 | 5 | 23.8 | 8 | 0 | 0 |
| 60–69 | 42 | 2 | 4.8 | 27 | 2 | 7.4 | 9 | 0 | 0 |
| ≧70 | 22 | 3 | 13.6 | 69 | 7 | 10.1 | 23 | 0 | 0 |
| Total | 221 | 43 | 12.9[b] | 141 | 17 | 12.1 | 49 | 0 | 0 |

[a]Melanoma, basal cell carcinoma, fibrosarcoma, Paget's disease.
[b]Adjusted to the age distribution of women with invasive squamous tumours.

**Table II** Proportion of women with squamous vulvar neoplasms who had coexisting condyloma, by year of diagnosis and county of residence

| | Stage of disease | | | | | |
|---|---|---|---|---|---|---|
| | In situ | | | *Invasive* | | |
| | *Total number* | *With condyloma Number* | *With condyloma Percent* | *Total number* | *With condyloma Number* | *With condyloma Percent* |
| *Year of diagnosis* | | | | | | |
| 1974–75 | 40 | 1 | 2.5 | 25 | 4 | 16.0 |
| 1976–77 | 54 | 10 | 18.5 | 36 | 2 | 5.6 |
| 1978–79 | 64 | 14 | 21.9 | 43 | 5 | 11.6 |
| 1980–81 | 63 | 18 | 28.6 | 37 | 6 | 16.2 |
| *County of residence* | | | | | | |
| Urban[a] | 182 | 37 | 20.3 | 102 | 14 | 13.7 |
| Rural | 39 | 6 | 15.4 | 39 | 3 | 7.7 |

[a]Three metropolitan counties with 75% of the total population.

possibility that some sort of selection process has led to an anomalously high proportion of women with warts. Second, for purposes of comparison, we have identified the prevalence of vulvar warts in a group of women who did not have squamous tumours.

An important limitation of our study relates to the fact that there was neither a standardized review of the accuracy of the diagnoses of the vulvar neoplasms, nor a standardized examination of cases and "controls" for the presence of vulvar warts. In order for our findings to have validity it is necessary to assume that, if vulvar warts are present in a woman with a vulvar neoplasm, their presence will be noted in the medical record and abstracted by the tumour registrar with the same diligence regardless of the neoplasm's histologic type. We believe this to be a reasonable assumption. Since a spectrum of atypia can occur in condylomata that can resemble squamous carcinoma *in situ* [Crum *et al.*, 1982], the failure to eliminate cases that were not truly malignant could have led to an artificially strong association. However, the finding of an elevated frequency of condylomata in women with invasive as well as *in situ* vulvar squamous tumours, as compared to women with nonsquamous tumours, argues that the association is real. Furthermore, the increased frequency of warts was found in patients diagnosed throughout the thirteen county area, not just in those residing in urban areas where the quality of pathologic diagnoses might be better.

The strong association between condylomata acuminata and vulvar squamous tumours is evidence in support of a causal relationship between the two. Even so, it does not imply a high absolute risk of vulvar squamous tumours for the woman with one or more condylomata. The combined incidence of vulvar *in situ* and invasive squamous tumours – less than 4 per 100,000 women per year [SEER, 1973–77] – gives rise to fewer than 4000 new cases in the United States annually, very much smaller than the number of women who develop condylomata each year in that country [MMWR, 1983]. The particular aspects of the condylomata, their treatment, or the woman that predispose to malignant change are poorly understood. The elucidation of these ought to be a high priority for subsequent research in this field.

We thank Anne Peterson for her assistance in the preparation of this manuscript. Availability of data from the Cancer Surveillance System, and the assistance of its staff, are greatly appreciated.

### References

BUSCEMA, J., WOODRUFF, J.P., PARMLEY, T.H. & GENADRY, R. (1980). Carcinoma *in situ* of the vulva. *Obstet. Gynecol.*, **55**, 225.

CRUM, C.P., FU, Y.S., LEVINE, R.U., RICHART, R.M., TOWNSEND, D.E. & PENGOLIO, C.M. (1982) Intraepithelial squamous lesions of the vulva: Biologic and histologic criteria for the distinction of condylomas from vulvar intraepithelial neoplasia. *Am. J. Obstet. Gynecol.*, **144**, 77.

FRIEDRICH, G., WILKINSON, E.H. & FU, Y.S. (1980). Carcinoma *in situ* of the vulva: A continuing challenge. *Am. J. Obstet. Gynecol.*, **136**, 830.

KOVI, J., TILLMAN, R.L. & LEE. S.M. (1974). Malignant transformation of condyloma acuminatum. *Am. J. Clin. Pathol.*, **61**, 702.

MANTEL, N. & HAENSZEL, W. (1959). Statistical aspects of the analysis of data from retrospective studies of disease. *J. Natl. Cancer Inst.*, **22**, 719.

MORBIDITY AND MORTALITY WEEKLY REPORT (1983). Condyloma Acuminatum – United States, 1966–1981. *MMWR*, **32**, 306.

RASTKAR, G., OKAGAKI, T., TWIGGS, L.B. & CLARK, B.S. (1982). Early invasive and *in situ* warty carcinoma of the vulva: Clinical, histologic, and electron microscopic study with particular reference to viral association. *Am. J. Obstet. Gynecol.*, **143**, 814.

SURVEILLANCE, EPIDEMIOLOGY, AND END RESULTS INCIDENCE AND MORTALITY DATA (1973–77). *Natl Cancer Inst. Monogr.*, **57**.

WOODRUFF, J.D., BRAUN, L., CAVALIERI, R., GUPTA, P., PASS, F. & SHAH, K.V. (1980). Immubologic Identification of papillomavirus antigen in condyloma tissues from the female genital tract. *Obstet. Gynecol.*, **56**, 727.